# The State of Standards in Genomic Sciences

**DOI:** 10.4650/sigs.2515706

**Published:** 2011-12-31

**Authors:** George M. Garrity

**Affiliations:** Department of Microbiology and Molecular Genetics, Michigan State University

On July 20, 2009, we began the public phase of an experiment in open access publishing with the first issue of *Standards in Genomic Sciences* (SIGS) [1]. The rational for the journal was to fulfill a perceived need in the community for the continued publication of “genome papers”, the once familiar companion articles that accompanied the public release of genome sequencing projects. Those papers served not only as a formal record of the accomplishment of the individuals involved in the sequencing and annotation efforts, but also provided the initial (and often the only) description of the sequence itself [2]. However, by 2007, Liolios *et al.* [3] had already pointed out that the publication of such papers significantly lagged behind the release of new genome sequences, leaving a gap in the public research record. Beyond genome reports, there was also a growing demand for other types of articles to meet the needs of a growing ‘omics community including detailed standard operating procedures that provide sufficient detail to not only understand the methods by which sequences were generated and annotated, but to also reproduce those results. Also needed was a reliable venue for publication of white papers and the proceedings of meetings of standards-setting bodies, such as the Genomic Standards Consortium (GSC) [4]. SIGS was conceived to fill these needs.

As 2011 draws to an end and we close out the final issue of Volume 5 of SIGS, we thought it appropriate to provide our authors, reviewers and readers with a brief update on the “state of the journal”, to examine the evidence that supports our original idea for the need for a journal such as SIGS, and to briefly outline key plans for the future.

## Milestones

One of the significant hurdles for any new publication is acceptance by potential authors and readers. Authors must be willing to take the risk of contributing articles to an untested journal and readers must be willing to take a risk reading and citing those articles in their own work. We have been fortunate in that SIGS became a primary outlet for articles derived from the *Genomic Encyclopedia of Bacteria and Archaea* (GEBA) [5]. Early on it became obvious that our highly structured and standardized Short Genome Report format was well suited for the project, as it would allow comparison of descriptive information about the genomes and the source organisms. In addition, adherence to the same format for genomes derived from other sequencing projects meant that readers could easily place genomes into a consistent and predictable framework. Similarly, reviewers could easily process manuscripts and spot discrepancies that might otherwise go unnoticed. The format has proven to be quite successful and in February 2011, the 100^th^ Short Genome Report was published in SIGS. An additional 50 Short Genome Reports were published by the end of the year. To date, all but one [6] of the Short Genome Reports was for a bacterial or archaeal genomes.

The taxonomic coverage of the Short Genome Reports published to date is presented in Table 1. Thus far, Short Genome Reports have been published for species or subspecies belonging to 16 of the 32 phyla containing types bearing validly published names.

**Table 1:** Sequenced bBacterial and archaeal type strains having sequenced genomes with and a companion publication

**Phylum**	**Type strains**	**SIGS**	**Other**	**Sequenced genomes**
***Archaea***				
*Crenarchaeota*	57	7	4	44
*Euryarchaeota*	316	11	11	169
*Thaumarchaeota*	1	0	1	2
***Bacteria***				
*Aquificae*	27	2	0	10
*Thermotogae*	38	0	1	16
*Thermodesulfobacteria*	7	0	0	3
*Deinococcus-Thermus*	71	5	0	24
*Chrysiogenetes*	4	1	0	3
*Chloroflexi*	25	4	1	16
*Nitrospirae*	9	0	0	2
*Deferribacteres*	12	3	0	9
*Cyanobacteria*	12	0	0	11
*Chlorobi*	12	0	0	9
*Proteobacteria*	3446	35	169	603
*Firmicutes*	1804	14	110	449
*Tenericutes*	202	0	14	39
*Actinobacteria*	2413	32	51	233
*Planctomycetes*	10	3	0	13
*Chlamydiae*	12	0	6	18
*Spirochaetes*	106	3	7	42
*Fibrobacteres*	2	0	1	2
*Acidobacteria*	12	0	0	3
*Bacteroidetes*	791	21	17	180
*Fusobacteria*	33	4	0	16
*Gemmatimonadetes*	1	0	0	6
*Verrucomicrobia*	33	1	4	6
*Dictyoglomi*	2	0	0	2
*Lentisphaerae*	2	0	1	2
*Synergistetes*	15	4	0	14
*Caldiserica*	1	0	0	1
*Elusimicrobia*	1	0	0	1
*Armatimonadetes*	1	0	0	0
**Total**	9478	150	398	1948

Totals are based on an export of the *Bacterial* and *Archaeal* taxonomic and nomenclatural events in the NamesforLife Database on December 30, 2011 [7]. There are 32 named phyla that are currently in common usage to which the validly named species and subspecies are mapped. Genome sequences are based on those that are declared as types from the GOLD database (5/28/2011) to which those genomes that were published outside of SIGS after that date were added. *Cyanobacteria* species are based on those species described in *Bergey’s Manual of Systematic Bacteriology*, Vol 1., 2nd Ed. 2001 and represent the dominant morphotypes. Non-redundant non-type strains, bearing validly published names, for which types have yet to be sequences are added to the table to minimize potential for overlap.

To better gauge progress of sequencing efforts in general, a new type of article was introduced in May 2011; a listing of genomes published outside of SIGS. The rationale for this article is to provide the community with a regularly updated list of sequenced genomes for which companion articles have been published. We were able to identify 397 of these articles that were published in 18 journals [8-11]. Excluding the genome sequences of viruses and eukaryotes, the taxonomic coverage of those papers differed somewhat from those published in SIGS, presumably because of the design of the GEBA project, which has focused on the genomes of taxonomic type strains available from public culture collections to maximize diversity.

**Figure 1:**
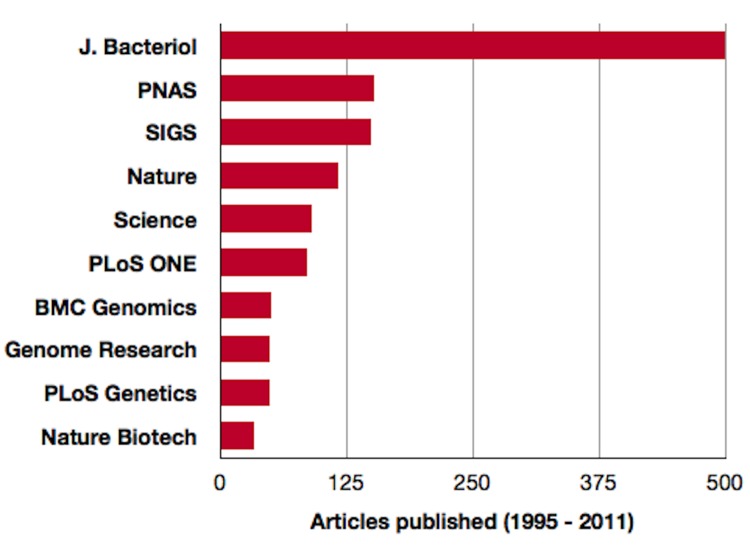
**Top ten journals in which genome publications have appeared**. To date approximately 1,518 articles have appeared in over 60 peer-reviewed publications. Source - Genomes Online Database and *Standards in Genomic Sciences*.

Nonetheless, the total number of genome articles remains relatively low (approximately 1,550) compared to the number of genome sequencing projects that either have been completed or are currently underway (11,221; GOLD). Coverage in the scientific literature tends to be somewhat sporadic and unpredictable, with reports appearing in more than 60 peer reviewed journals. However, more than 90% of genome reports have appeared in only ten journals (Figure 1). As of December 31, 2011, SIGS ranked third among the top ten periodicals publishing genome reports and will likely move into the second position during the first quarter of 2012. A breakdown of the articles published in SIGS, by type, is presented in Table 2.

**Table 2:** Articles published in Standards in Genomic Sciences, Vol 1-5

**Category of** **Articles**	**Number of Articles**
Community dialog	10
Editorial	5
Erratum	2
Genome table	4
Meeting report	15
Research article	6
Short genome report	149
Short metagenome report	1
Standard Operating Procedure	10
White Paper	2
**Total**	203

Other notable events in 2011 include a special issue dedicated to meeting reports and community dialog articles by various standards groups (Vol 5 No 2) and the first effective publication of a taxonomic proposal for a new bacterial species [12] published in SIGS. We also published our first short genome report for a virus [6] and a white paper from the zoological community advocating for sequencing the genome of the garter snake [13].

## Community Acceptance

There are several indications that SIGS has been well received by the scientific community. In February 2011, the journal became part of the PubMed Central (PMC) open access collection. All of the SIGS content has been deposited in the PMC archive and is available in HTML, PDF and XML form. Listing in PMC has resulted in an increase in our readership, with approximately one third of our readers accessing content from the PMC site. SIGS has also been integrated into the larger body of scientific literature. During the second and third quarter of 2011, we were informed that SIGS would be included in the Scopus (Elsevier) and Web of Science (Thomson-Reuters) indices.

**Figure 2:**
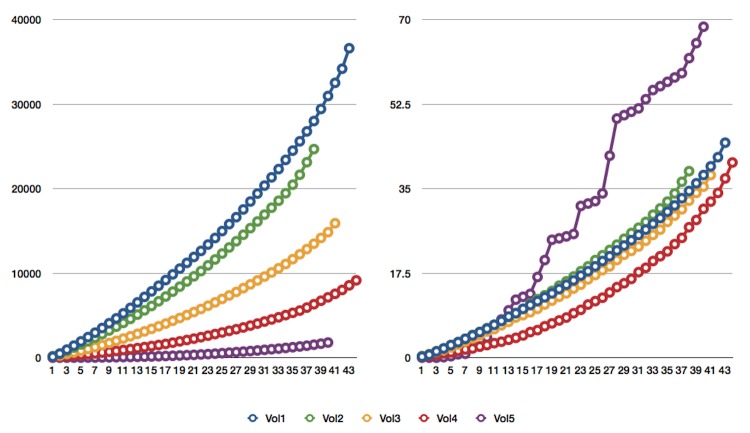
**Combined download statistics from the journal site and PubMed Central for articles published in *Standards in Genomic Sciences*, by volume**. Left panel – total cumulative downloads since initial publication on the Internet. Right panel, cumulative daily downloads of articles. X-axis – number of articles per volume. Y-axis – number of downloads.

Web traffic has continued to increase steadily (Table 3). At the end of 2011, we were experiencing 230 downloads of articles/day on the SIGS site and 121 downloads/day on the PMC site. The total number of article downloads since SIGS began publishing is rapidly approaching 120,000 (Figure 2). Daily downloads tend to be high for each issue for the first several weeks after publishing on either site, after which the download frequency tends to decline. However, we have not yet observed a plateau for any of the published volumes as we continue to have new visitors on the site each day. This suggests that we have not yet saturated the potential audience. The top five articles downloaded from the SIGS and PMC site are listed in Table 4.

**Table 3:** Key web traffic statistics

SIGS home page		
	Daily downloads	230
	Total downloads	88,250
PMC page		
	Daily downloads	121
	Total downloads	30,268
Traffic source		
	Cities	4,377
	Countries	152

**Table 4:** Top five papers from the SIGS and PubMed Central sites based on total and daily downloads

**Authors**	**Title**	**Down- loads**
**SIGS site - total**		
Hallin, *et al*.	GeneWiz browser: An Interactive Tool for Visualizing Sequenced Chromosomes [14].	2426
Sims *et al*.	Complete genome sequence of *Kytococcus sedentarius* type strain (541^T^) [15].	1667
Mavromatis *et al*.	The DOE-JGI Standard Operating Procedure for the Annotations of Microbial Genomes [16].	1560
Snipen *et al*.	Standard operating procedure for computing pangenome trees [17].	1550
Lapidus *et al*.	Complete genome sequence of *Brachybacterium faecium* type strain (Schefferle 6-10^T^) [18].	1533
**SIGS site - daily**		
Bini *et al*.	Complete genome sequence of *Desulfurispirillum indicum* strain S5^T^ [19].	7.8
Nelson and Garrity	Genome sequences published outside of Standards in Genomic Sciences, December 2011 [11].	7.8
Copeland *et al*.	Complete genome sequence of the halophilic and highly halotolerant *Chromohalobacter salexigens* type strain (1H11^T^) [20].	5.8
Schleheck *et al*.	Complete genome sequence of *Parvibaculum lavamentivorans* type strain (DS-1^T^) [21].	4.1
Humann *et al*.	Complete genome of the onion pathogen *Enterobacter cloacae* EcWSU1 [22].	3.5
**PMC site - total**		
Gilbert *et al*.	The Earth Microbiome Project: Meeting report of the “1st EMP meeting on sample selection and acquisition” at Argonne National Laboratory October 6th 2010 [23].	758
Gilbert *et al*.	Meeting Report: The Terabase Metagenomics Workshop and the Vision of an Earth Microbiome Project [24].	715
Tanenbaum *et al*.	The JCVI standard operating procedure for annotating prokaryotic metagenomic shotgun sequencing data [25].	494
Gilbert	Metagenomes and metatranscriptomes from the L4 long-term coastal monitoring station in the Western English Channel [26].	435
Snipen and Ussery	Standard operating procedure for computing pangenome trees. [17].	405
**PMC site - daily**		
Lorenzi *et al*.	The Viral MetaGenome Annotation Pipeline (VMGAP):an automated tool for the functional annotation of viral Metagenomic shotgun sequencing data [27].	2.9
Gilbert *et al*.	The Earth Microbiome Project: Meeting report of the “1st EMP meeting on sample selection and acquisition” at Argonne National Laboratory October 6th 2010 [23].	2.4
Gilbert *et al*.	Meeting Report: The Terabase Metagenomics Workshop and the Vision of an Earth Microbiome Project [24].	2.3
Anderson *et al*.	Complete genome sequence of the hyperthermophilic chemolithoautotroph *Pyrolobus fumarii* type strain (1A^T^) [28].	2.0
Castoe *et al*.	A proposal to sequence the genome of a garter snake (*Thamnophis sirtalis*) [13].	1.8

Download statistics for the SIGS site were generated using Google Analytics. Daily reads were estimated based on the number of days from the time an issue appeared online to December 31, 2011 (range 8 – 881 days). Statistics for the PMC site were collected estimate based on data reported by the PMC Publisher Services site from the day an article was posted to December 31, 2011.

Our reader community also continues to grow. Article downloads on the SIGS and PMC sites map to 15,350 unique IP addresses located in 4,377 cities in 152 countries. Although SIGS has not been publishing long enough to estimate our impact factor, 93 articles have been cited a total of 271 times in articles included in the Cite-by-Linking program of Cross-Ref.

## Moving forward

Our experience with the template for Short Genome Reports has been largely successful. The layout of content is highly predictable and simplifies writing, reviewing, editing and reading these articles. Yet, we are exploring the possibility of some minor changes to the tabular layout in 2012, to accommodate an anticipated influx of articles from the Thousand Genome Project, which represents the second phase of the GEBA initiative. Although it is unlikely that we will be able to “auto-generate” manuscripts as a part of the sequencing and annotation pipeline, this represents an early attempt to capture and standardize much of the summarized data that is incorporated into Short Genome Reports. This will also give us an opportunity to explore how to more tightly integrate the literature and databases.

The second major change for 2012 deals with funding SIGS in the future. We were very fortunate in that seed funding for SIGS was provided through grants from the Office of the Vice President of Research and Graduate Studies of Michigan State University and the Office of Biological and Environmental Research of the US Department of Energy. This has provided us with the opportunity to underwrite the publication costs of articles appearing in Volumes 1 – 4 and a limited number of articles in Volume 5. However, like other open access publications we need to institute a cost recovery mechanism to sustain publication of SIGS. More information about the publication fees is included in the Instructions to Authors.
